# No effect of adding dairy lipids or long chain polyunsaturated fatty acids on formula tolerance and growth in full term infants: a randomized controlled trial

**DOI:** 10.1186/s12887-018-0985-2

**Published:** 2018-01-22

**Authors:** Maria Lorella Gianni, Paola Roggero, Charlotte Baudry, Catherine Fressange-Mazda, Pascale le Ruyet, Fabio Mosca

**Affiliations:** 1 Neonatal Intensive Care Unit (NICU), Department of Clinical Science and Community Health, Fondazione IRCCS “Ca’ Granda” Ospedale Maggiore Policlinico, University of Milan, Milan, Italy; 2 Nutrition Department, Lactalis R&D, Retiers, France; 3 Lactalis Nutrition Europe, Torcé, France

**Keywords:** Infant formula, Lipid quality, Dairy lipids, Dairy fat, Fatty acids, Growth, Body composition, Fat mass, Gastrointestinal tolerance, Regurgitation

## Abstract

**Background:**

When breastfeeding is not possible, infants are fed formulas in which lipids are usually of plant origin. However, the use of dairy fat in combination with plant oils enables a lipid profile in formula closer to breast milk in terms of fatty acid composition, triglyceride structure and cholesterol content. The objectives of this study were to investigate the impact on growth and gastrointestinal tolerance of a formula containing a mix of dairy lipids and plant oils in healthy infants.

**Methods:**

This study was a monocentric, double-blind, controlled, randomized trial. Healthy term infants aged less than 3 weeks whose mothers did not breastfeed were randomly allocated to formula containing either: a mix of plant oils and dairy fat (D), only plant oils (P) or plant oils supplemented with long-chain polyunsaturated fatty acids (PDHA). Breastfed infants were included in a reference group (BF). Anthropometric parameters and body composition were measured after 2 and 4 months. Gastrointestinal tolerance was evaluated during 2 day-periods after 1 and 3 months thanks to descriptive parameters reported by parents. Nonrandomized BF infants were not included in the statistical analysis.

**Results:**

Eighty eight formula-fed and 29 BF infants were enrolled. Gains of weight, recumbent length, cranial circumference and fat mass were similar between the 3 formula-fed groups at 2 and 4 months and close to those of BF. Z-scores for weight, recumbent length and cranial circumference in all groups were within normal ranges for growth standards. No significant differences were noted among the 3 formula groups in gastrointestinal parameters (stool frequency/consistency/color), occurrence of gastrointestinal symptoms (abdominal pain, flatulence, regurgitation) or infant’s behavior.

**Conclusions:**

A formula containing a mix of dairy lipids and plant oils enables a normal growth in healthy newborns. This formula is well tolerated and does not lead to abnormal gastrointestinal symptoms. Consequently, reintroduction of dairy lipids could represent an interesting strategy to improve lipid quality in infant formulas.

**Trial registration:**

ClinicalTrials.gov Identifier NCT01611649, retrospectively registered on May 25, 2012.

## Background

Breastfeeding is considered by the World Health Organization (WHO) as the best choice for infant feeding. Human milk composition is used as a guideline for establishing minimum and maximum levels of nutrients in formulas in order to meet infant’s needs. Lipids are major components in human milk, providing 45–55% of total energy intake. They fulfill various metabolic and physiological functions critical for the development, growth and health of the newborn [1]. Human milk contains a wide variety of lipid species, present as milk fat globules with a core containing triacylglycerols (more than 98% of total lipids), surrounded by a milk fat globule membrane with complex lipids (phospholipids and sphingolipids), esterified cholesterol, proteins and glycoproteins. Human milk provides alpha-linolenic (ALA) and linoleic acids (LA) which are essential fatty acids (EFA). They can be endogenously converted by the newborn into long-chain derivatives of Omega 3 and Omega 6 families, such as docosahexaenoic acid (DHA) and arachidonic acid (ARA), respectively [2]. However, this conversion is considered as low in humans. Human milk also contains preformed DHA and ARA, at levels strongly influenced by the mother’s diet. These fatty acids (FA) constitute the main components of the brain and they have an important impact on neuronal and visual functions [3].

Fat sources used in most infant formulas currently marketed are a mixture of plant oils. Indeed, since the middle of twentieth century, infant formulas have been enriched in EFA-rich plant oils and bovine milk fat has been progressively removed. Infant formulas can also be optionally supplemented with fish/algae/single cell oils providing preformed DHA and ARA. However, lipids of plant oils are not comparable to lipids of human milk in terms of FA diversity, triacylglycerol structure, fat globule composition, complex lipids and cholesterol contents. Consequently, infant formulas provide milk-specific FA and cholesterol only when dairy lipids are used as a fat source in combination with plant oils [1]. Also, the nature of fat sources used in formulas strongly influences their FA composition. In particular, short and medium chain FA, lauric acid, myristic acid and palmitic acid contents can vary considerably according to the use of palm oil, coco oil or dairy lipids [1]. Moreover, plant oils do not possess the specific triglyceride structure found in breast milk or cow’s milk with palmitic acid in sn2 position. The triglyceride structure is of particular importance because long-chain saturated FA in the sn2 position are more efficiently digested and absorbed [4].

Dairy lipids specifically contain about 10% of myristic acid and 10% of short and medium chain FA (C4-C10) while plant oils provide much less of these FA. Short chain FA represent a rapid source of energy for the infant, because they enter directly the portal vein and are known to be completely absorbed and oxidized. Myristic and short/medium chain FA could also increase bioavailability and conversion of n-3 PUFA [5]. Also, it was shown in adults that a moderate intake of dairy lipids and rapeseed oil improved DHA levels in erythrocytes and blood fluidity [6, 7]. Furthermore, recent studies in omega 3-deficient rodents have demonstrated that for a similar ALA content, a blend of dairy lipids and rapeseed oil induced a higher level of brain DHA than a blend of plant oils even if supplemented with preformed DHA [8, 9].

Consequently, dairy lipids could represent an interesting strategy to improve lipid composition of infant formula in order to better mimic human milk composition and structure and to optimize growth and health of the infant. In this study, we evaluated the impact on growth as well as the tolerance to a formula containing a blend of dairy lipids and plant oils during the first 4 months of life.

## Methods

### Study design

This monocentric, double-blind, controlled, randomized trial was conducted in 2012–2013 in the Department of Neonatology of the Fondazione IRCCS Cà Granda Ospedale Maggiore Policlinico, Milano, Italy (NCT01611649). The study was approved by the local Ethical Committee and conducted in accordance with Good Clinical Practice and the principles and rules of the Declaration of Helsinki. Parents or legal caregivers provided written informed consent prior to enrollment of their infants in the study. The study protocol has been previously published [10].

### Population

Healthy full-term infants born in the Department of Neonatology were screened for participation in the study. When breastfeeding was not possible (contraindication or mothers not intended to breastfeed), infants were randomly allocated to be fed for 4 months with a formula containing either: a mix of plant oils and dairy fat (D), only plant oils (P) or plant oils supplemented with ARA and DHA (PDHA). Infants whose mothers intended to exclusively breastfeed from birth through at least 4 months were enrolled in a nonrandomized reference group (BF). Inclusion criteria were: gestational age 37 to 42 weeks, birth weight > 2500 g, healthy newborns from normal pregnancy, aged up to 3 weeks when entering the study, no breastfeeding (for the formula-fed groups) or exclusive breastfeeding (for the reference group). Exclusion criteria were: positive family history of allergy to milk proteins, known congenital or postnatal diseases which could interfere with the study and newborns whose parents had planned to move within 6 months after birth.

### Study formulas

Study formulas were formulated into powders and were reconstituted at 13.3%. They were manufactured and provided by Milumel®, Lactalis, Craon, France. The 3 tested formulas were packaged in blinded containers labeled only with study details and number of randomization; they were indistinguishable in appearance and texture. The randomization schedule was computer-generated and stratified on sex. Sequentially numbered tins of infant formula were prepared according to this schedule. Once the newborn was enrolled, he/she was allocated to the next available randomization number which corresponded to the allocation to one of the 3 study formulas. Both the investigators and the infants’ parents were blind to the group allocation. Compositions of the 3 study formulas were in compliance with the European Directive 2006/141/EC on infant formulas and are detailed in Table 1 (manufacturer data). The 3 formulas had similar energy and macronutrient contents but they differed by the nature of their lipid sources. Formula D contained a mixture of dairy lipids and plant oils; formula P contained only plant oils and formula PDHA contained plant oils supplemented with ARA and DHA. Study formulas were consumed straightaway after randomization and were provided for the 4 subsequent months.

**Table 1:** Composition of study formulas

		Formula D	Formula P	Formula PDHA
		100 ml^a^	100 ml^a^	100 ml^a^
Energy	kJ	275	275	275
	kcal	66	66	66
Protein	g	1.3	1.3	1.3
Carbohydrates	g	8.1	8.1	8.1
Lactose	g	6.8	6.8	6.8
Fat	g	3.1	3.1	3.1
LA	mg	439	549	549
	% TFA	14.2	17.7	17.7
ALA	mg	73	55	55
	% TFA	2.3	1.8	1.8
Ratio LA/ALA		6	10	10
ARA	mg			12.4
	% TFA			0.4
DHA	mg			6.2
	% TFA			0.2
Ratio ARA/DHA				2

^a^Reconstituted 13.3%

Composition of tested formulas according to manufacturer data. Formula D contained a mixture of dairy lipids and plant oils; formula P contained only plant oils and formula PDHA contained plant oils supplemented with ARA and DHA. *LA* linoleic acid, *ALA* alpha-linolenic acid, *ARA* arachidonic acid, *DHA* docosahexaenoic acid, *TFA* total fatty acids

#### Objectives and outcomes

The main objective of this study was to investigate the effect of formula D on the erythrocyte membrane Omega 3 fatty acid content as compared to formulas P and PDHA [10]. The secondary objective was to evaluate the safety of infants consuming formula D in comparison to infants consuming formula P and PDHA and to breastfed infants. Therefore, the impact of formulas on growth, body composition and gastrointestinal tolerance was assessed and only these results are reported in this article.

#### Evaluation of growth, body composition and gastrointestinal tolerance

Baseline data (sex, gestational age at birth, mode of delivery and anthropometric measurements) were recorded at enrollment. Growth and body composition were evaluated at enrollment and after 2 and 4 months of consumption of the allocated formula (or breastfeeding) during follow-up visits. The infants’ anthropometric measurements (body weight, length, head circumference) were obtained using standardized techniques. Subject mass was measured on an electronic scale accurate to the nearest 0.1 g. Recumbent length was measured on a Harpenden stadiometer to the nearest 1 mm. The head circumference was measured using a non-stretch measuring tape to the nearest 1 mm. Z-scores for weight, length and head circumference were then calculated with WHO Anthro for personal computers, version 3.2.2, 2011 (http://www.who.int/childgrowth/software/en/). Body composition was assessed using an air displacement plethysmography system (PEA POD Infant Body Composition System, COSMED- USA). A detailed description of the PEA POD’s physical design, operating principles, validation and measurement procedures is provided elsewhere [11, 12]. The PEA POD assesses fat mass and fat free mass by direct measurements of body mass and volume and the application of classic densitometric principles.

Gastrointestinal (GI) tolerability was evaluated thanks to diaries filled by parents for 2-day periods before a phone call planned after 1 and 3 months of consumption of the allocated formula (or breastfeeding). Specifically, the following indicators of GI tolerability were collected through multiple-choice questions: volume of formula intake, daily frequency of stool passage, stool consistency and color, episodes and type of regurgitations (no regurgitation/regurgitations of small amounts/regurgitations of large amounts/vomits), episodes of flatulence and abdominal pain (defined as intermittent attacks of abdominal pain when the baby screams and draws up his/her legs but is well between episodes). Sleeping disturbances and general behavior were also described. Adverse events were recorded throughout the study period.

#### Sample size

Calculation of sample size for this study has been previously detailed [10].

#### Statistical analyses

Statistical analyses were performed using SAS software (SAS Institute Inc., Cary NC, USA) by Soladis, Lyon, France. All statistical analyses were performed on an intention to treat basis. Continuous variables were expressed as mean and standard deviation. Differences among groups for growth parameters were analyzed with an analysis of variance with 3 fixed factors (formula, sex, time). Differences among groups for GI parameters were analyzed with ordinal or binary logistic model on formula and time effects with age as covariate. A *p*-value < 0.05 was considered significant. As the breastfed infants were not randomized, no statistical analysis was performed to compare the breastfed group with any of the formula-fed groups.

## Results

### Study population

**Fig. 1:**
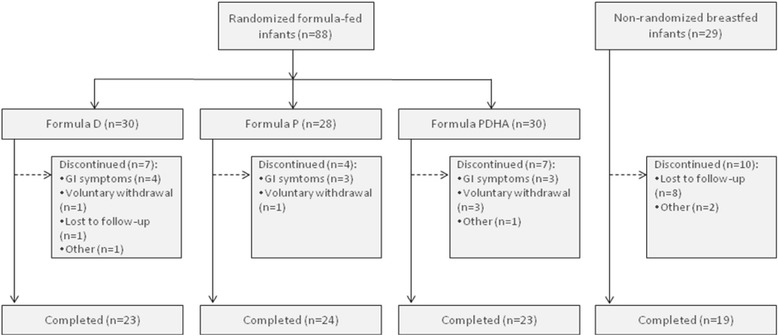
Flow of study subjects. Formula D contained a mixture of dairy lipids and plant oils; formula P contained only plant oils and formula PDHA contained plant oils supplemented with ARA and DHA. GI (gastrointestinal) symptoms included regurgitations, reflux, constipation, abdominal pain and flatulence

A total of 117 healthy newborns were enrolled. Of these, 88 were randomized to the formula-fed groups and 29 were enrolled in the breastfed reference group (Fig. 1). A total of 18 (20%) infants from the formula groups and 10 (34%) in the breastfed group withdrew before the end of the study (Fig. 1). Rates of discontinuation were similar in the 3 formula groups (23% in formula D and PDHA; 14% in formula P). GI symptoms (ie. regurgitations, reflux, constipation, abdominal pain and flatulence) were the most common reason for study discontinuation in the 3 formula groups: 57% in group D, 75% in group P and 43% in group PDHA. Among the breastfed group, 8 babies were lost to follow-up and breastfeeding was stopped before the next visit for 2 babies.

Baseline characteristics in each group are presented in Table 2. Ethnicity was not recorded in this study. Proportion of boys was similar in the 3 formula groups (53–57%; *p* = 0.851) and was of 38% in the breastfed reference group (Table 2). A high proportion of caesarean births (43–64%) was observed in all groups without any difference among the 3 formula groups (*p* = 0.185). Gestational age was around 38 weeks in the 3 formula groups (*p* = 0.970) and around 39 weeks in the breastfed group. On average, infants were enrolled in the study at 5–10 days of life (Table 2). Age at inclusion was significantly different in the 3 formula groups (*p* = 0.016) with a lower age in group P than in group PDHA (4.9 ± 4.5 vs. 9.6 ± 7.9 days; *p* = 0.012). Weight, recumbent length, head circumference and body composition were similar in the 3 formula groups at baseline (all *p* > 0.05) (Table 2).

**Table 2:** Infants’ characteristics at enrollment

		Formula D	Formula P	Formula PDHA	Breastfed	*p*-value
		*n* = 30	*n* = 28	*n* = 30	*n* = 29	
Sex, no. (%)	Girls	13 (43.3)	11 (39.3)	14 (46.7)	18 (62.1)	*0.851*
	Boys	17 (56.7)	17 (60.7)	16 (53.3)	11 (37.9)	
Delivery mode, no. (%)	Natural	11 (36.7)	10 (35.7)	17 (56.7)	14 (48.3)	*0.185*
	Caesarean	19 (63.3)	18 (64.3)	13 (43.3)	15 (51.7)	
Gestational age, weeks, mean (SD)		38.4 (1.2)	38.4 (1.1)	38.3 (1.0)	39.0 (1.0)	*0.970*
Age at enrollment, days, mean (SD)		6.8 (5.6)	4.9 (4.5) ^X^	9.6 (7.9)	8.8 (6.6)	0.016*
Weight, g, mean (SD)		3144.2 (448.6)	2942.9 (386.6)	3156.4 (431.8)	3308.7 (533.9)	*0.107*
Recumbent length, cm, mean (SD)		49.1 (2.2)	48.9 (2.2)	49.7 (1.9)	50.1 (1.8)	*0.351*
Head circumference, cm, mean (SD)		34.4 (1.2)	34.3 (1.4)	34.7 (1.4)	35.3 (2.0)	*0.476*
Fat mass FM, mean (SD)	% BW	10.3 (2.9)	10.8 (3.4)	9.8 (3.5)	11.6 (5.4)	*0.487*
Fat-free mass FFM, mean (SD)	% BW	89.7 (2.9)	89.2 (3.4)	90.2 (3.5)	88.4 (13.8)	*0.487*

Formula D contained a mixture of dairy lipids and plant oils; formula P contained only plant oils and formula PDHA contained plant oils supplemented with ARA and DHA. *FM* fat mass, *FFM* fat-free mass, *BW* body weight. Comparison of the 3 formula groups by ANOVA 1 fixed factor (Formula); * *p* < 0.05; ^X^ *p* < 0.05 vs. PDHA

Mean daily volume of formula consumed was evaluated during a 2 day-period at 1 month and 3 months. Most of formula-fed infants consumed more than 600 ml daily at 1 month (around 95% of infants) and more than 700 ml daily at 3 months (around 82%). No significant differences in formula intake were observed among the 3 formula groups at 1 month (*p* = 0.980) or at 3 months (*p* = 0.177).

### Growth

Mean weight gains in formula groups were 1.9–2.2 kg at 2 months (33–36 g/day) and 3.4–3.8 kg at 4 months (27–31 g/day). In breastfed infants, weight gain was 2.1 kg at 2 months (33 g/day) and 3.3 kg at 4 months (28 g/day). No differences of daily weight gains were observed among the 3 formula groups (*p* = 0.211) (Table 3). Mean daily gains in length were not significantly different among the 3 groups despite a tendency (*p* = 0.055). In particular, length gain tended to be lower with formula P than with formula D and PDHA (Table 3). Overall, gains in length were significantly lower in girls than in boys (p = 0.05). Finally, daily gains in head circumference were similar in the 3 formula groups (*p* = 0.082).

**Table 3:** Growth rates between baseline and 2 or 4 months of age

		Formula D	Formula P	Formula PDHA	Breastfed	*p*-value
n	2 mo	23	25	24	22	
	4 mo	23	24	23	19	
Weight gain, g/day, mean (SD)	2 mo	34.0 (7.1)	33.0 (9.7)	36.2 (6.8)	32.8 (7.7)	*0.211*
	4 mo	30.0 (5.1)	27.3 (5.0)	31.3 (5.3)	27.6 (6.8)	
Length gain, mm/day, mean (SD)	2 mo	1.34 (0.33)	1.22 (0.38)	1.35 (0.33)	1.38 (0.35)	*0.055*
	4 mo	1.16 (0.18)	1.05 (0.17)	1.17 (0.15)	1.06 (0.17)	
Head circumference gain, mm/day, mean (SD)	2 mo	0.78 (0.13)	0.71 (0.20)	0.78 (0.19)	0.65 (0.19)	*0.082*
	4 mo	0.62 (0.08)	0.57 (0.09)	0.64 (0.08)	0.54 (0.15)	

Formula D contained a mixture of dairy lipids and plant oils; formula P contained only plant oils and formula PDHA contained plant oils supplemented with ARA and DHA. Comparison of the 3 formula group by ANOVA 3 fixed factors (Sex, Time, Formula and all interactions)

**Fig. 2:**
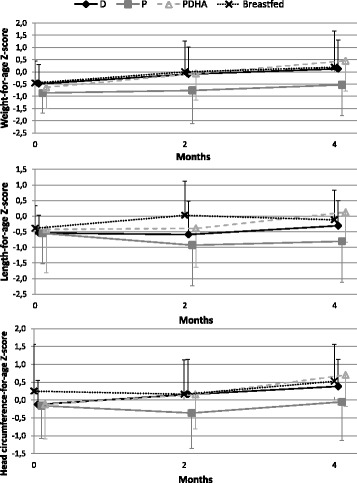
Mean growth measurements of infants relative to WHO growth standards. Formula D contained a mixture of dairy lipids and plant oils; formula P contained only plant oils and formula PDHA contained plant oils supplemented with ARA and DHA. WHO: World Health Organization

Compared with WHO growth standards, infants in all groups grew normally throughout the study. Mean values for all growth measures through 4 months of age were within 1 SD of the WHO median values (Fig. 2).

**Fig. 3:**
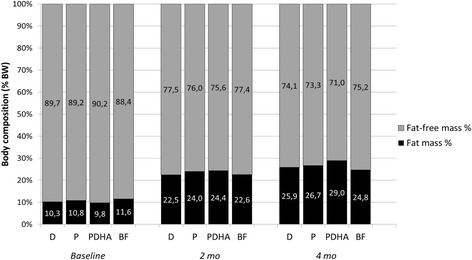
Body composition expressed in percentage of body weight at baseline, 2 months and 4 months in formula-fed and breastfed infants. Formula D contained a mixture of dairy lipids and plant oils; formula P contained only plant oils and formula PDHA contained plant oils supplemented with ARA and DHA. BW: body weight. Comparison of the 3 formula group by ANOVA 3 fixed factors (Sex, Time, Formula and all interactions)

Body composition was similar in the 3 formula-fed groups after 2 and 4 months (Fig. 3). Fat mass significantly increased over time (*p* < 0.001) and was significantly higher in girls than in boys (*p* = 0.008) without any difference among the 3 formula-fed groups (*p* = 0.489) (Fig. 3).

### GI tolerability

**Fig. 4:**
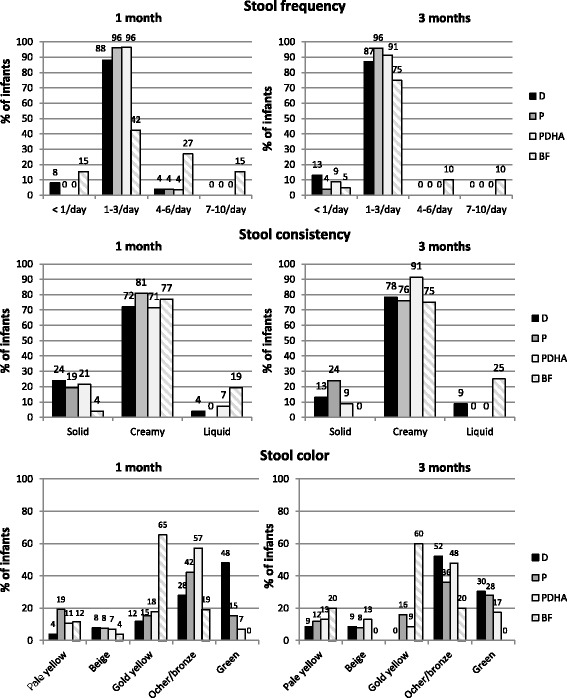
Infants’ stool frequency, consistency and color at 1 and 3 months of age. Formula D contained a mixture of dairy lipids and plant oils; formula P contained only plant oils and formula PDHA contained plant oils supplemented with ARA and DHA. BF: breastfed. Comparison of the 3 formula group by ordinal logistic model on formula and time effects with age as covariate

Daily stool frequency was comprised between 1 and 3 stools per day for the majority of formula-fed infants at 1 month (88–96%) and 3 months of age (87–96%) (Fig. 4). At 1 month, one infant in each formula group had a stool frequency of 4–6 stools/day and 2 infants in the formula D group had less than 1 stool/day (Fig. 4). At 3 months, no formula-fed infant had 4–6 stools/day but some had less than 1 stool/day (3 in formula D, 1 in formula P and 2 in PDHA). No significant difference was observed among the 3 formula groups for daily stool frequency (*p* = 0.335). In the breastfed group, daily stool frequency distribution was more equally spread among classes, with 42% and 75% of infants with 1–3 stools per day at 1 and 3 months, respectively; 27% and 10% with 4–6 stools/day; 15% and 10% with 7–10 stools/day and 15% and 5% with less than 1 stool/day (Fig. 4). Stool consistency was mainly creamy in formula-fed infants at 1 month (71–81%) and 3 months (76–91%), sometimes solid but rarely liquid (Fig. 4). No significant difference was observed among the 3 formula groups for stool consistency (*p* = 0.486). Breastfed infants also had mainly creamy stools (75–77%) but they were more likely to have liquid but not solid ones (Fig. 4). Stool color was mainly green in formula D-fed infants at 1 month (48%) while it was mainly ocher/bronze in groups P and PDHA (42 and 57% respectively). At 3 months, stools were mainly ocher/bronze for the 3 formula groups (Fig. 4). Stools of breastfed infants were mainly gold yellow or ocher/bronze but never green (Fig. 4).

No significant difference was reported among the 3 formula in caregivers’ reports of abdominal pain (*p* = 0.823) which was more frequent at 1 month (27–44% of infants) than at 3 month (9–13% of infants; *p* = 0.02). In the breastfed group, abdominal pain was reported in 54 and 80% of infants at 1 and 3 months, respectively (Table 4). Reports of flatulence were similar in the 3 groups (*p* = 0.125) and they were more frequent at 1 month (68–85% of infants) than at 3 months (57–68%; *p* = 0.001). In the breastfed group, flatulence affected 65 and 60% of infants, respectively (Table 4). Sleeping disturbances were observed in 8–25% of formula-fed infants at 1 month and 4–24% of infants at 3 months, without statistical differences among groups (*p* = 0.276). General behavior was described similarly in the 3 formula groups (*p* = 0.651) (Table 4).

**Table 4:** Occurrence of abdominal pain, flatulence and sleeping disturbances and description of behavior in infants at 1 and 3 months of age

		1 month								3 months							
		Formula D *n* = 25		Formula P *n* = 26		Formula PDHA *n* = 28		Breastfed *n* = 26		Formula D *n* = 23		Formula P *n* = 25		Formula PDHA *n* = 23		Breastfed *n* = 20	
		No.	%	No.	%	No.	%	No.	%	No.	%	No.	%	No.	%	No.	%
Abdominal pain		11	44	7	27	11	39	14	54	2	9	3	12	3	13	16	80
Flatulence		17	68	22	85	21	75	17	65	13	57	17	68	13	57	12	60
Sleeping disturbances		2	8	4	15	7	25	10	38	1	4	6	24	1	4	4	20
Behavior	Quiet	17	68	15	58	18	64	20	77	15	65	12	48	11	48	7	35
	Active	8	32	9	35	7	25	5	19	8	35	13	52	11	48	13	65
	Disturbed/restless	0	0	2	8	3	11	1	4	0	0	0	0	1	4	0	0

Formula D contained a mixture of dairy lipids and plant oils; formula P contained only plant oils and formula PDHA contained plant oils supplemented with ARA and DHA. Comparison of the 3 formula group by binary logistic model on formula and time effects with age as covariate

**Fig. 5:**
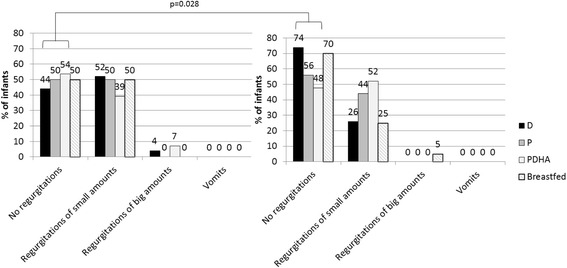
Description of regurgitations and vomits in infants at 1 (left) and 3 months (right). Formula D contained a mixture of dairy lipids and plant oils; formula P contained only plant oils and formula PDHA contained plant oils supplemented with ARA and DHA. Comparison of the 3 formula group by binary logistic model on formula and time effects with age as covariate

At 1 month, regurgitations of small amounts of formula or breast milk after the meal were frequent in all groups (around 50% of infants) without any difference among groups (*p* = 0.742). Between 1 and 3 months, frequency of regurgitations decreased significantly (*p* = 0.028) without any difference among groups (Fig. 5). No vomit was described and regurgitations of big amounts were reported only in 1 formula D-fed and 2 formula P-fed infants at 1 month and in 1 breastfed infant at 3 months (Fig. 5).

## Discussion

This study demonstrated that 4-month consumption of a formula containing dairy lipids was associated with normal growth in healthy term infants and was well tolerated. There were no statistically significant differences in weight, length, or head circumference growth rates among groups fed with formula containing either a mix of dairy and plant lipids, or a blend of plant oils (with or without ARA and DHA). Long Chain PUFA addition had no effect on growth. In this study, growth rates of formula-fed were close to those of breastfed babies. Furthermore, weight, length and head circumference measurements during the 4 first months of life in all groups were similar to the WHO growth standards.

One limitation of this study is that sample size was not initially calculated for the specific outcomes presented here, in particular weight gain. According to the guidance provided by the American Academy of Pediatrics [13], the number of subjects of a specified sex needed in each group to detect a 3 g/day difference in weight gain over a 3.5 month-period with a power of 0.8 in a one- tailed test is 28. Consequently, this study might be underpowered to detect a statistical difference in weight gain. Body composition was also similar in the 3 formula groups. It has been previously described that formula-fed had different body composition compared to breastfed babies, with higher fat-free mass (in grams) at 4 months and higher fat-free mass changes between enrollment and 4 months [14]. However, in this previous study, fat mass and fat-free mass at 4 months (both expressed in % of body weight) were similar between breastfed and formula-fed babies [14]. In our study, fat mass and fat-free mass values in 4 month-old formula-fed infants were close to those described by Gianni et al., 2014 (around 27% and 73% of body weight, respectively). Fat-free mass change after 4 months was also lower in breastfed infants (+ 62%) than in formula-fed babies (+ 74–83%). These results suggest that formula-fed infants show different body composition development during the first 4 months of life compared to breastfed babies. However, the nature of fat used in the tested formulas did not significantly affect body composition.

No significant differences were noted among the 3 formula groups in gastrointestinal parameters (stool frequency, consistency or color), occurrence of gastrointestinal symptoms (abdominal pain, flatulence, regurgitation or vomiting) or infant’s behavior. Stool frequency tended to be higher in breastfed than formula-fed babies (with 42% and 20% of breastfed infants at 1 month at 3 months, respectively, having more than 4 stools/day versus 4 and 0% of formula-fed ones). This result is in agreement with previous data showing that breastfed infants pass stools almost 50% more often than formula-fed ones [15]. Moreover, solid stools were observed more often in formula-fed than in breastfed babies (19–24% at 1 month and 9–24% at 3 months in formula-fed versus 4 and 0% in breastfed). It has been previously reported that hard stools are associated with formula feeding and are related to the presence of excreted fatty acid soaps and calcium [16]. Indeed, palmitic acid from plant oils is differentially arranged in the triglyceride (mainly in sn1 and sn3 position, only 5–20% in sn2 position also called beta-palmitate) from palmitic acid from breast milk (60% of beta-palmitate). Palmitic acid from plant oils is poorly absorbed and can react with calcium to form insoluble soaps leading to decreased fat and calcium absorption and increased stool hardness [17]. In dairy lipids, around 45% of palmitic acid is esterified in sn2 position [18]. In infant formula, this results in a lower proportion of beta-palmitate in formulas containing only vegetable oils compared with formulas containing dairy lipids or structured lipids [19]. In our study, the formula containing dairy lipids contained more beta-palmitate than formulas with blends of plant oils. However, no difference was observed among these formula on the occurrence of solid stools. This could be due to methodological considerations with a low number of babies in each group and the inaccuracy of stool consistency report (only 2 time points with evaluation on the last 2 days). Further investigation would be required especially to evaluate if the nature of fat in formula has an impact on fat content in stools.

A high frequency of gastrointestinal symptoms was present throughout the study, especially regurgitations which affected around 50% of babies at 1 month and around 30% of babies at 3 months, without any difference among groups. However, regurgitation can be considered as a physiological phenomenon in the first year of life and these frequencies are in accordance with previous data reporting that more than 50% of infants aged 0–5 months regurgitate at least once a day [20]. Intermittent episodes of abdominal pain were also observed in 27–44% of formula-fed infants at 1 month and 9–13% at 3 months, which is slightly higher than in the usual range of incidence for colic [21]. However, in our study, the incidence of colic, as defined by Wessel [22], was not evaluated. Therefore, the tolerability and safety of a formula containing a mix of dairy and plant lipids did not appear to be different from formulas containing blend of plant oils with or without ARA and DHA.

## Conclusions

A formula containing dairy lipid provides adequate nutrition for normal growth in healthy term infants and is as well tolerated as control formulas containing only plant oils as lipid sources. Long Chain PUFA addition in a plant oil-based formula has no effect on growth and formula tolerance. Breast milk remains the food of choice but for infants who cannot be breastfed, this study shows that a formula containing dairy lipids having a fat profile closer to breast milk is an appropriate alternative. Further research is needed to investigate the impact of a dairy lipid-containing formula on growth of low birth weight preterm infants as well as its health benefits both in term and preterm infants.

## Abbreviations

ALA: Alpha-linolenic acid
ARA: Arachidonic acid
BF: Breastfed
BW: Body weight
DHA: Docosahexaenoic acid
EFA: Essential fatty acid
FA: Fatty acid
FFM: Fat-free mass
FM: Fat mass
formula D: with a mix of plant oils and dairy lipids
formula P: with only plant oils
formula PDHA: with plant oils and supplemented with ARA and DHA
GI: Gastrointestinal
LA: Linoleic acid
PUFA: Polyunsaturated fatty acid
SD: Standard deviation
TFA: Total fatty acids
WHO: World Health Organization
